# A panel of four genes accurately differentiates benign from malignant thyroid nodules

**DOI:** 10.1186/s13046-016-0447-3

**Published:** 2016-10-28

**Authors:** Qing-Xuan Wang, En-Dong Chen, Ye-Feng Cai, Quan Li, Yi-Xiang Jin, Wen-Xu Jin, Ying-Hao Wang, Zhou-Ci Zheng, Lu Xue, Ou-Chen Wang, Xiao-Hua Zhang

**Affiliations:** 1 Department of Oncology, The First Affiliated Hospital of Wenzhou Medical University, Wenzhou, Zhejiang Province 325000 China; 2 Department of Otolaryngology Head and Neck Surgery, Xinhua Hospital, Shanghai Jiaotong University, School of Medicine, Shanghai, 200000 China

**Keywords:** Thyroid nodules, Diagnostic panel, Biomarkers

## Abstract

**Background:**

Clinicians are confronted with an increasing number of patients with thyroid nodules. Reliable preoperative diagnosis of thyroid nodules remains a challenge because of inconclusive cytological examination of fine-needle aspiration biopsies. Although molecular analysis of thyroid tissue has shown promise as a diagnostic tool in recent years, it has not been successfully applied in routine clinical use, particularly in Chinese patients.

**Methods:**

Whole-transcriptome sequencing of 19 primary papillary thyroid cancer (PTC) samples and matched adjacent normal thyroid tissue (NT) samples were performed. Bioinformatics analysis was carried out to identify candidate diagnostic genes. Then, RT-qPCR was performed to evaluate these candidate genes, and four genes were finally selected. Based on these four genes, diagnostic algorithm was developed (training set: 100 thyroid cancer (TC) and 65 benign thyroid lesions (BTL)) and validated (independent set: 123 TC and 81 BTL) using the support vector machine (SVM) approach.

**Results:**

We discovered four genes, namely fibronectin 1 (FN1), gamma-aminobutyric acid type A receptor beta 2 subunit (GABRB2), neuronal guanine nucleotide exchange factor (NGEF) and high-mobility group AT-hook 2 (HMGA2). A SVM model with these four genes performed with 97.0 % sensitivity, 93.8 % specificity, 96.0 % positive predictive value (PPV), and 95.3 % negative predictive value (NPV) in training set. For additional independent validation, it also showed good performance (92.7 % sensitivity, 90.1 % specificity, 93.4 % PPV, and 89.0 % NPV).

**Conclusions:**

Our diagnostic panel can accurately distinguish benign from malignant thyroid nodules using a simple and affordable method, which may have daily clinical application in the near future.

**Electronic supplementary material:**

The online version of this article (doi:10.1186/s13046-016-0447-3) contains supplementary material, which is available to authorized users.

## Background

Thyroid cancer (TC) is the most common endocrine malignancy [1]. Recently, the number of thyroid carcinoma cases annually had increased by 4 % globally [2] and became the fastest growing type of cancer in many countries [1, 3]. China also suffers a large burden from thyroid cancer [4, 5]. China accounts for a large portion of thyroid cancer patients around the world. Papillary thyroid cancer (PTC) accounts for 80–85 % of all types of thyroid carcinomas [6].

As a result of the increased frequency of medical examinations, such as ultrasonic examination, clinicians are confronted with an increasing number of patients with thyroid nodules. However, the majority of these thyroid nodules are benign, and only a few cases are malignant [7, 8]. Therefore, accurately diagnosing thyroid nodules preoperatively is important for patients with thyroid cancer to receive timely treatment appropriately. Also, those with benign thyroid nodules can avoid unnecessary treatments, such as diagnostic surgery. Preoperative diagnosis of thyroid nodules can also help to reduce medical burden of patients and the government to a large extent, particularly in developing countries.

Cytological examination by ultrasound-guided fine-needle aspiration biopsy (US-FNAB) is the most accurate and gold-standard preoperative diagnostic method currently [9, 10]. However, about 17 % (10–26 %) of FNAB are reported as indeterminate, and 6 % (1–11 %) were nondiagnostic; while 72 % median (range 62–85 %) of undertaken FNAB were benign, and 5 % (1–8 %) were malignant [11]. As a consequence, most of these patients with indeterminate result undergo unnecessary diagnostic surgery, making them suffer from lifelong replacement therapy of thyroid hormones and corresponding surgical complications [12].

In recent years, molecular analysis of thyroid tissue has shown promise as a diagnostic tool [8, 13–15]. A microarray-based test, combining the expression levels of 167 genes (The Afirma) can diagnose thyroid nodules with 92 % sensitivity and 52 % specificity [13]. Another panel, named ThyroSeq v2 assay, which consists of more than 50 gene mutations and gene fusions (more than 1,000 hotspots), makes accurate diagnosis with 90 % sensitivity, 93 % specificity, 83 % positive predictive value (PPV), and 96 % negative predictive value (NPV) by using next-generation sequence [8]. Other studies also performed with impressive PPV or NPV [14–17]. Nevertheless, studies aforementioned have their limitations. Some studies contain large amounts of gene alterations, such as The Afirma and ThyroSeq v2 assay, which are expensive and difficult to promote, particularly in less-developed countries. Some studies failed to show both optimal sensitivity and specificity in validation analysis. Moreover, majority of these panels were based on patients from Western countries. Since genetic background is different between Asians and Western populations, whether these panels could be successfully applied to Chinese patients still remain unknown.

Therefore, in the present study, we performed whole-transcriptome sequencing of 19 paired-PTC tissue samples and bioinformatics analysis to investigate global mRNA expression. By using molecular approaches and statistical models, we successfully established a diagnosis model that combines only four genes: fibronectin 1 (FN1), gamma-aminobutyric acid type A receptor beta 2 subunit (GABRB2), neuronal guanine nucleotide exchange factor (NGEF), and high-mobility group AT-hook 2 (HMGA2). This diagnosis model could discriminate malignant from benign thyroid nodules with both high sensitivity and specificity in Chinese patients.

## Methods

### Next generation sequencing and bioinformatics analysis

Total RNA was extracted from tissue samples using TRIZOL Reagent (Invitrogen) according to the manufacturer’s protocol. After the quality test, The cDNA libraries were prepared using Ion Total RNA-Seq Kit v2.0 (Life Technologies) according to the manufacturer’s instructions. The cDNA libraries were then processed for sequencing using Illumina Hiseq 2500 according to the commercially available protocols. Before reads mapping, clean reads were obtained from the raw reads by removing the adaptor sequences, reads with > 5 % ambiguous bases, and low-quality reads. The clean reads were then aligned to the human genome (version: GRCH37) using the MapSplice program (v2.1.6, University of Kentucky, Lexington, KY, USA). We applied Ebseq algorithm to screen out the differently expressed genes using the following criteria: 1) fold change (FC) > 2 for up or downregulation; 2) false discovery rate (FDR) < 0.05 and 3) *P*-Value < 0.05.

### Patients and tissue collection

Primary TC samples or benign thyroid lesions and their matched adjacent normal thyroid tissue samples were obtained at the time of initial surgery from the First Affiliated Hospital of Wenzhou Medical University. Inclusion and exclusion criteria are as follows, inclusion criteria: patients with surgical indication; tumor size larger than 5 mm; papillary thyroid cancer or follicular thyroid cancer or anaplastic thyroid cancer or medullary thyroid cancer or benign thyroid lesions; with accurate postoperative pathological diagnosis; with informed consent from patients. Exclusion criteria: tumor size less than or equal to 5 mm; with inconclusive postoperative pathological diagnosis; with other malignant tumors; patients with surgical contraindications. Samples were snap-frozen in liquid nitrogen immediately after surgical resection and subsequently stored at a −80 °C freezer. All the training and validation samples set used in this study are snap frozen tissues. Histopathological slides were reviewed retrospectively for all cases to confirm the histological diagnosis and to ensure abundant cancer content of the tumor by two pathologists. Detailed information about these patients are shown in Additional files 1 and 2. Informed consent for the scientific use of biological material was obtained from each patient and the work was approved by the Ethics Committee of the First Affiliated Hospital of Wenzhou Medical University.

### RNA isolation and RT-qPCR

Total RNA was isolated using TRIZOL Reagent (Invitrogen) and reverse transcription (TOYOBO, Japan) was performed according to the manufacturer’s instructions. Quantitative real-time PCR (RT-qPCR) analysis was performed in triplicate on the ABI prism 7500 sequence detection system (Applied Biosystems, USA) using the THUNDERBIRD SYBR qPCR Mix (TOYOBO, Japan) according to manufacturer’s instructions. The GAPDH mRNA level was used for normalization. Primer sequences were as follows: FN1: 5′- TGGCCCCCACCTTCTTG -3′ (forward) and 5′- TGCGGGAAAAATCCCTTCTA -3′ (reverse); GABRB2: 5′- ACTGCCGTGTCACATCCTCTAA -3′ (forward) and 5′- TGGAACTGTCAACTTGCTTCAAA -3′ (reverse); NGEF: 5′- GAGCTGGAAATGGTGGTGAAG -3′ (forward) and 5′- TTCCGTGCGGCTCATTTT -3′ (reverse); HMGA2: 5′- GACCCAAAGGCAGCAAAAAC -3′(forward) and 5′- AGTGGCTTCTGCTTTCTTTTGAG -3′(reverse); GAPDH: 5′-GGTCGGAGTCAACGGATTTG-3′(forward) and 5′-ATGAGCCCCAGCCTTCTCCAT -3′(reverse).

### Model development

We evaluated several methodologies for building statistical models that can predict benign versus malignant thyroid nodules based on expression level of the four genes. Finally, the SVM (support vector machine) was used for classification. With the modeling platform of MATLAB 2014a, we establish the SVM model for the classification of benign and malignant thyroid nodules by using Libsvm 3.20. During the development of SVM model, using the training data, we chose the C-SVC and RBF kernel functions and used the grid search method to debug the model. The grid c bound was −8 to 8, the grid c step was 0.5, the grid g bound was −8 to 8, and the grid g step was 0.5. The fold number of cross validation was 5.

$$ \mathrm{Plabel}=\mathrm{s}\mathrm{g}\mathrm{n}\left(\Sigma \mathrm{n}\mathrm{i}=0\ \mathrm{w}\mathrm{i}\  \exp \left(\hbox{-} \mathrm{gamma}\left|\ \left(\mathrm{xi}\hbox{-} \mathrm{x}\right)\right|2+\mathrm{b}\right)\right) $$

### Statistical analysis

Data on normal distribution were expressed as mean ± standard variation (SD) and were compared with t-test. Categorical variables were expressed as percentage and were compared with Chi-square test or Fisher’s exact test, as appropriate. All *P* values were two sided, and a *P* value of < 0.05 was considered statistically significant. Statistical analysis was performed with SPSS software version 19.0 (SPSS, Chicago, IL, USA). GraphPad Prism 5 (GraphPad Software, USA) was used for graphs.

## Results

### Filtrating differentially-expressed genes in TC by whole-transcriptome sequence

**Fig. 1:**
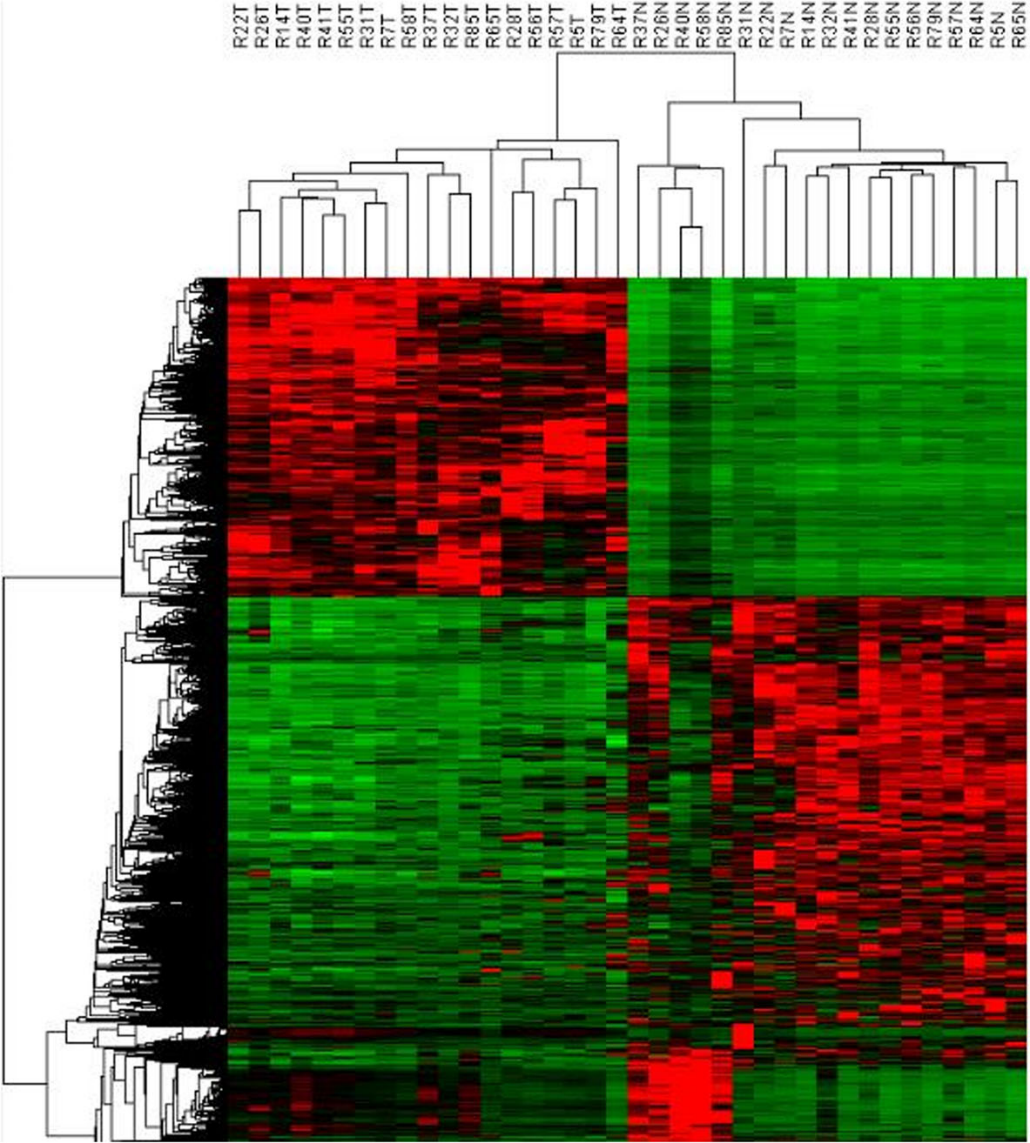
Differentially expressed mRNAs in PTC tissue samples and adjacent normal tissue samples are analyzed using hierarchical clustering. Each row represents a single mRNA and each column represents one tissue sample. *Red* indicates high relative expression and *green* indicates low relative expression

**Fig. 2:**
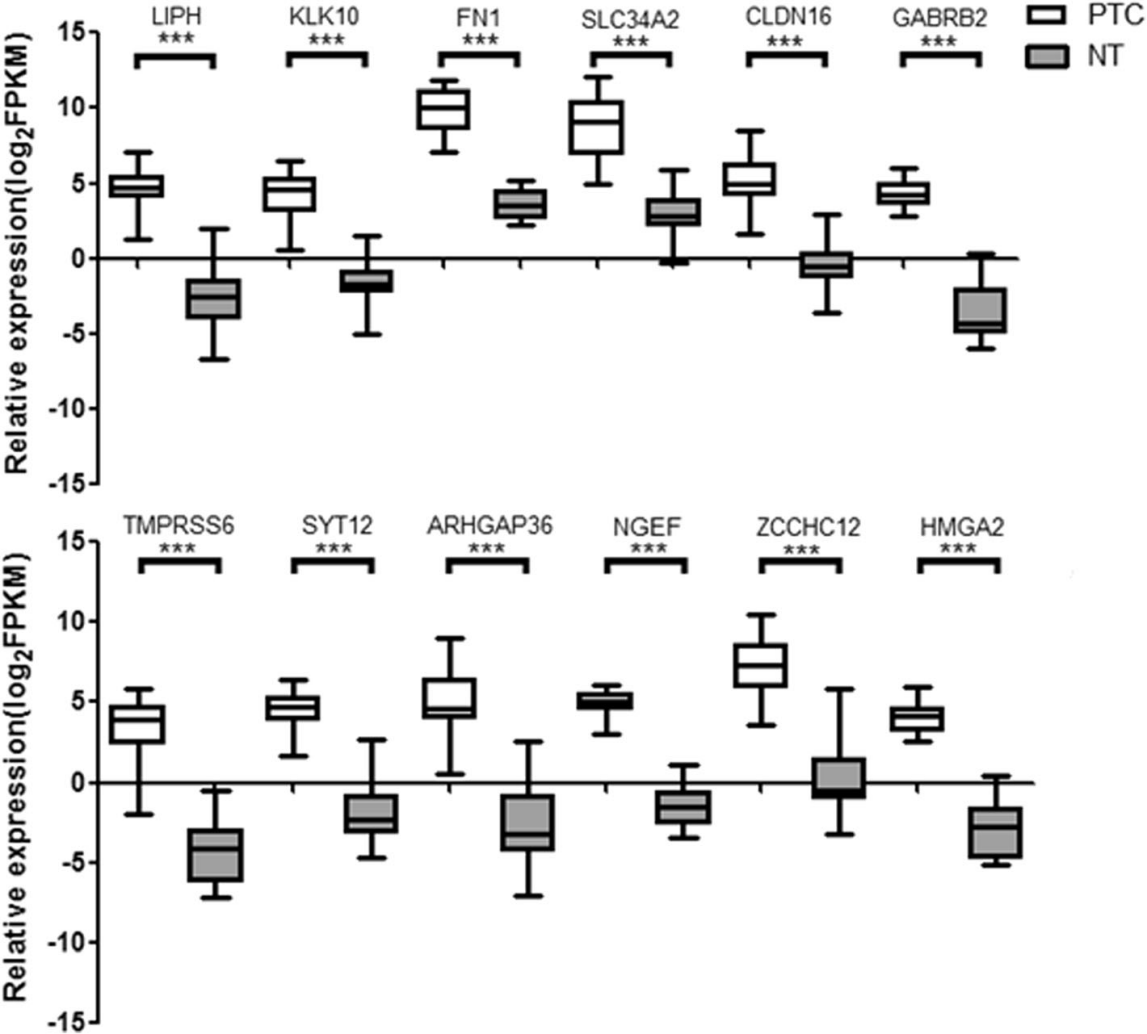
Expression of the 12 overexpressed genes in our sequencing data (19 PTC and 19 NT). **P < 0.05; ***P < 0.001

**Fig. 3:**
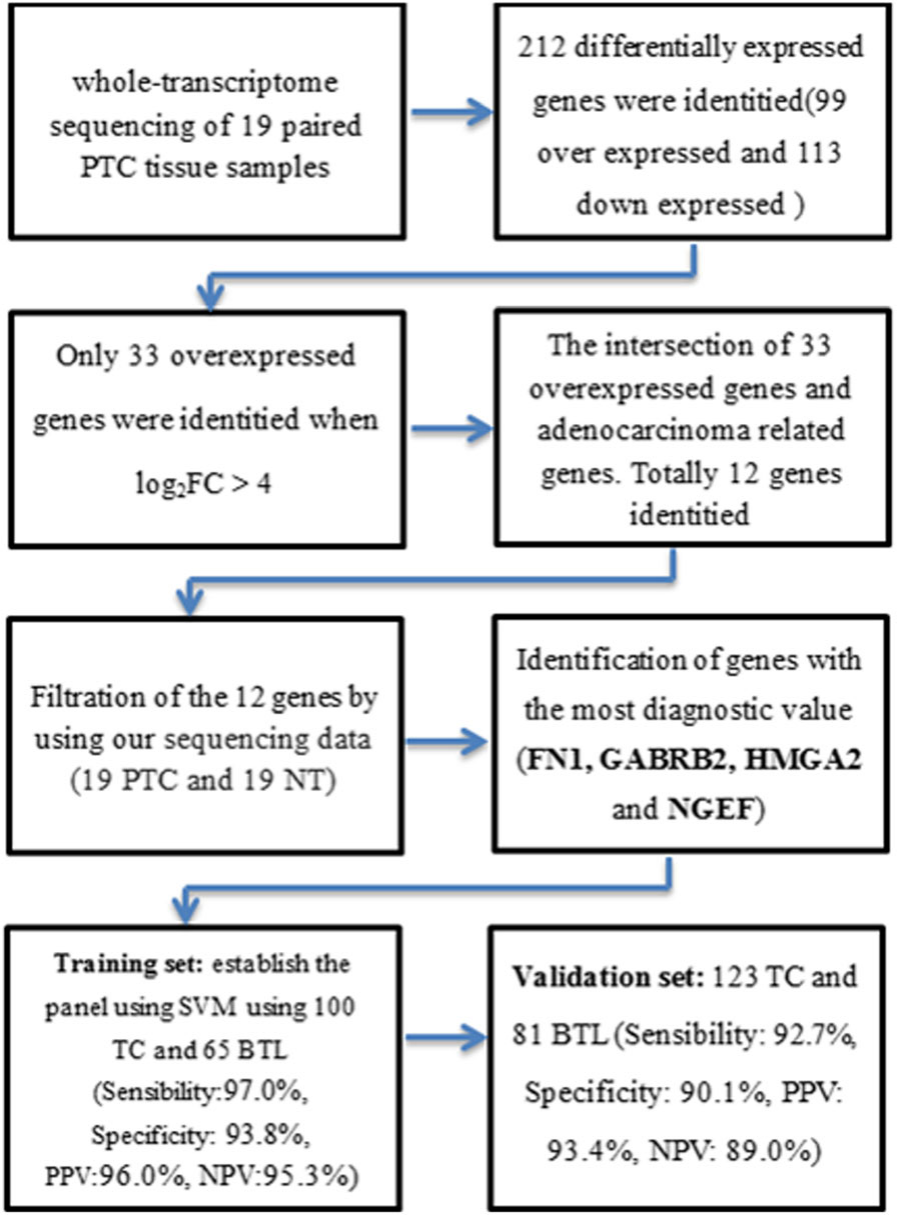
Flowchart of the study design. Totally 212 differential expressed genes were identitied by using whole-transcriptome sequencing of 19 paired PTC tissue samples. Only 33 genes were identitied when log_2_FC > 4 and we only focusd on those 33 over expressed genes. Then we got the intersection of those 33 over expressed genes and adenocarcinoma related genes, including 12 genes. we investigated the expression of these 12 overexpressed genes in our 19 paired PTC samples with sequencing data, 4 genes with the most diagnostic value were picked out (FN1, GABRB2, NGEF and HMGA2). High accuracy of diagnosis was both realized in training set and validation set

We performed whole-transcriptome sequencing of 19 paired-PTC tissue samples to filtrate differentially-expressed genes. We identified a total of 212 differentially-expressed genes from the 19 paired-PTC tissue samples (log_2_FC > 1.0, FDR < 0.05 and *P*-Value < 0.05) (Fig. 1), including 99 overexpressed and 113 downregulated genes, and then the 99 overexpressed genes were given focus. We raised the FC cut-off value to a higher level (log_2_FC > 4.0) to obtain more limited genes, which are significantly overexpressed. Only 33 overexpressed genes were identified. To obtain genes that are highly associated with thyroid tumor, we screened out the intersection of these 33 overexpressed genes and adenocarcinoma related genes, including only 12 genes (Fig. 2). Then, we investigated the expression of these 12 overexpressed genes in our 19 paired PTC samples with sequencing data (19 PTC and 19 NT). As shown in Fig. 2, all 12 genes showed significant differential expression between TC and NT, with FN1, GABRB2, NGEF and HMGA2 showing the best diagnostic value. Finally, these four genes with the most diagnostic value were filtrated. The flowchart of our study design is presented in Fig. 3.

### Training set: establishing the diagnostic panel

**Fig. 4:**
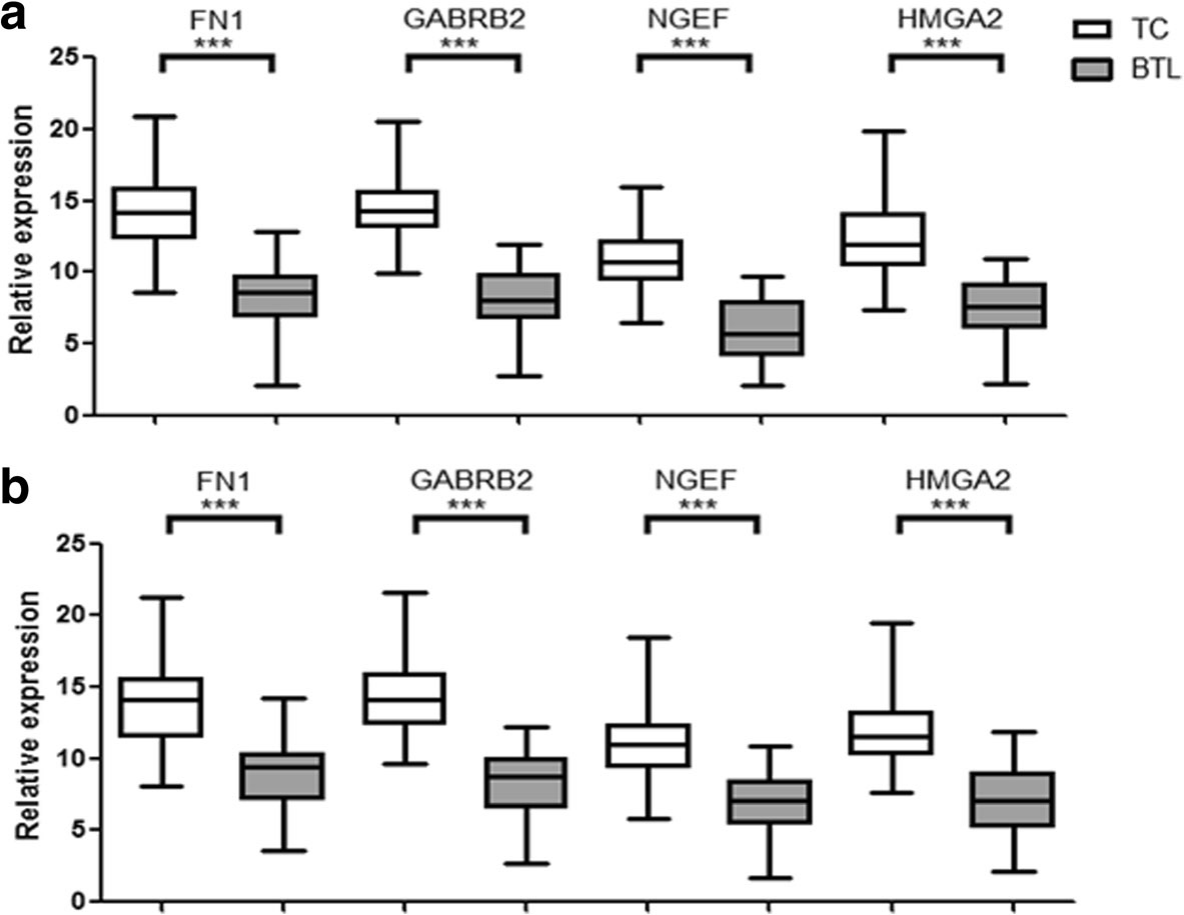
RT-qPCR analysis of the four genes. **a**. RT-qPCR analysis of the four genes in training set (100 TC and 65 BTL). **b**. RT-qPCR analysis of the four genes in validation set (123 TC and 81 BTL). **P < 0.05; ***P < 0.001

To establish diagnostic panel, mRNA expression levels of the four genes were assessed in 165 samples (100 TC and 65 BTL) using RT-qPCR. Result of each gene is shown in Fig. 4a. Each of four genes demonstrated decent ability to differentiate malignancy and benignity. Using SVM statistical model, we successfully established diagnostic panel that combines the four genes (FN1, GABRB2, NGEF and HMGA2). Results revealed that the panel was able to differentiate thyroid nodules excellently. 95.8 % (158/165 samples correctly classified) of the samples were correctly classified (Sensibility: 97.0 %, Specificity: 93.8 %, PPV: 96.0 %, NPV: 95.3 %).

### Validation set: validating the panel in external independent samples

To validate panel performance, mRNA expression levels of the four genes were measured by RT-qPCR in another 204 samples (123 TC and 81 BTL). Similar to previous data, relative expression level of the four genes were significantly upregulated (Fig. 4b). The panel also achieved high accuracy in validation set, with 91.7 % accuracy (187/204 samples correctly classified). Only nine malignant thyroid tumors (4.4 %) and 8 benign thyroid lesions (3.9 %) were misclassified among all the 204 samples. Panel performance based on RT-qPCR was satisfactory, with 91.7 % sensitivity, 92.7 % sensitivity, 90.1 % specificity, 93.4 % PPV and 89.0 % NPV.

## Discussion

Thyroid cancer, particularly PTC, has become the fastest growing type of cancer in recent years [1, 3]. Owing to the frequency of medical examinations, clinicians are now faced with an even increasing number of patients with thyroid nodules, most of which, however, are benign [7, 8]. Avoiding unnecessary surgery, especially total thyroidectomies, thereby reducing surgery-associated risks and subsequent treatments, is still an intractable challenge for clinicians. Thus, accurately distinguishing benign from malignant thyroid nodules preoperatively is important.

Considering this challenge, previous studies have developed several diagnostic panels through high-throughput technologies, like next-generation sequence and microarray [8, 13–15, 18, 19]. A prospective study evaluated diagnostic molecular test consisting of 167 genes in indeterminate nodules, which presented high sensitivity (92 %), but low specificity (52 %) [13]. Some additional research reports consistent results in using the classifier [20, 21], while some reported only 17 % malignancy rate confirmation in indeterminate nodules [22]. Moreover, the test is not cost-effective outside of North America [20]. Another panel, named ThyroSeq v2 assay, which consists of more than 50 gene mutations and gene fusions (more than 1,000 hotspots), performed with 90 % sensitivity, 93 % specificity, 83 % PPV, and 96 % NPV by using next-generation sequence [8]. Other studies also achieved encouraging effects [14–19]. However, these diagnostic panels are too complex and costly to apply in clinical practice. Also, they have not yet been proven to be effective in Asian population. Many studies have shown that genetic background is different between Asians and Western populations. For example, a gene expression classifier (Afirma) showed distinctly different sensitivity and specificity in different populations partly because of different genetic background [13, 20, 22–25]. What is more, Xing M et al. reported BRAF mutation occurred in about 45 % of PTC patients in the USA [26] while the rate reported in Asian was much higher, 63.7 % in the study from Jin L et al. [27] and 58 % in the study from Lee JH [28]. However, the rate of TERT promoter mutation was much higher in Western populations than Asian population (about 10 % vs 4.1 %) [27, 29]. Moreover, the incidence of thyroid cancer is also different between Asians and Western populations [3, 4].

In the present study, we developed a panel that presents excellent performance in discriminating benign from malignant thyroid nodules by combining only four genes (FN1, GABRB2, NGEF and HMGA2). Its performance was further validated by independent external samples. To further confirm its performance in FNAB samples, we also tested the panel in 20 FNAB samples with indeterminate result, which correctly recognized 17 of 20 with 85 % prediction accuracy. In spite of the limited sample size, the panel also showed good discriminating ability in FNAB samples.

FN1 encodes fibronectin involved in cell adhesion and migration processes, including embryogenesis, wound healing, blood coagulation, host defense and metastasis. This gene was reported to be differentially expressed in human cancers [30]. Some research showed that FN1 could help distinguish benign from malignant thyroid tumors [31, 32], which is consistent with our findings.

GABRB2 encodes gamma-aminobutyric acid A receptor, beta 2 subunit. This gene was found to be related to mental diseases, like schizophrenia [33, 34]. A recent study revealed that GABRB2 is highly upregulated in thyroid cancer [35]. In our study, GABRB2 was one of the most significant upregulated genes in 203 differential expression genes.

NGEF has been found to be associated with myopia and obesity-related diseases [36, 37]. However, it is not yet well studied in human cancer. A study from Korea had demonstrated that NGEF is a differentially-expressed gene in PTC [38]. We also found that NGEF is upregulated and can be used for diagnosis.

HMGA2 encodes protein that belongs to non-histone chromosomal high mobility group (HMG) protein family. This protein contains structural DNA-binding domains and may act as a transcriptional regulating factor. Many studies have confirmed that HMGA2 is important in tumorigenesis and tumor progression [39–43], including thyroid cancer [35, 44]. Chiappetta et al. reported that the mRNA expression of HMGA2 is associated with malignant phenotype in human thyroid neoplasias [44]. HMGA2 was also used as a diagnostic marker in previous studies [14, 45]. In our study, HMGA2 was significantly upregulated in primary TC tissue, compared with benign and normal thyroid nodules.

Although we had successfully established a diagnostic panel with excellent performance, the current study has several limitations. First, based on frozen tissue samples, our panel was only tested in a small FNAB size, so it needs to be validated in FNAB samples with large-sample size. Second, our study was a single-center study; multi-center prospective studies are needed.

## Conclusions

In summary, this study successfully established a diagnostic panel by using a simple and affordable method, which utilized only four genes with excellent performance in Chinese patients. We believe this panel could form the basis for a robust, practical, and affordable molecular diagnostic tool for clinical use in the near future.

## Additional files

Additional file 1: Table S1. Clinicopathologic characteristics of 223 thyroid cancer patients enrolled in the study. (DOCX 16 kb)
Additional file 2: Table S2. Clinicopathologic characteristics of 146 benign thyroid lesions patients enrolled in the study. (DOCX 14 kb)
