# Erratum to: A panel of four genes accurately differentiates benign from malignant thyroid nodules

**DOI:** 10.1186/s13046-017-0488-2

**Published:** 2017-01-24

**Authors:** Qing-Xuan Wang, Ou-Chen Wang, En-Dong Chen, Ye-Feng Cai, Quan Li, Yi-Xiang Jin, Wen-Xu Jin, Ying-Hao Wang, Zhou-Ci Zheng, Lu Xue, Ou-Chen Wang, Xiao-Hua Zhang

**Affiliations:** 1 Department of Oncology, The First Affiliated Hospital of Wenzhou Medical University, Wenzhou, Zhejiang, Province 325000 China; 2 Department of Otolaryngology Head and Neck Surgery, Xinhua Hospital, Shanghai Jiaotong University, School of Medicine, Shanghai, 200000 China

## Erratum

n.b. The errors and associated corrections described in this document concerning the original manuscript were accountable to the production department handling this manuscript, and thus are no fault of the authors of this paper.

During the production process Ou-Chen Wang was omitted from the list of corresponding authors in the original article [1]. Both Xiao-Hua Zhang and Ou-Chen Wang are corresponding authors of this article.
