# Supplementary material for: A panel of four genes accurately differentiates benign from malignant thyroid nodules
Source: J Exp Clin Cancer Res. 2016 Oct 28;35:169. doi: 10.1186/s13046-016-0447-3 (PMC5084448; doi:10.1186/s13046-016-0447-3)
Supplement: Additional file 1: Table S1. — Clinicopathologic characteristics of 223 thyroid cancer patients enrolled in the study. (DOCX 16 kb) [file 13046_2016_447_MOESM1_ESM.docx]

|  | | |
| --- | --- | --- |
| Supplemental Table 1. Clinicopathologic characteristics of 223 thyroid cancer patients enrolled in the study. | |  |
| Characteristics | No. Patients (%) |  |
| Category |  |  |
| PTC | 221(99.10%) |  |
| FTC | 2(0.90%) |  |
| ATC | 0(0%) |  |
| Age at diagnosis, y |  |  |
| Mean±SD | 48.09 ± 11.95 |  |
| < 45 y | 93(41.70%) |  |
| ≥ 45 y | 130(58.30%) |  |
| Gender |  |  |
| Female | 175(78.48%) |  |
| Male | 48(21.52%) |  |
| Tumor size in mm |  |  |
| Mean±SD | 13.92 ± 8.36 |  |
| ≤ 10mm | 101(45.29%) |  |
| > 10mm | 122(54.71%) |  |
| Hashimoto’s thyroiditis | 68(30.49%) |  |
| Multifocal or Unifocal |  |  |
| Multifocal | 70(31.39%) |  |
| Unifocal | 153(68.61%) |  |
| Perineural invasion |  |  |
| YES | 7(3.14%) |  |
| NO | 216(96.86%) |  |
| Extrathyroidal invasion |  |  |
| YES | 18(8.07%) |  |
| NO | 205(91.93%) |  |
| Lymph node metastasis |  |  |
| YES | 125(56.05%) |  |
| NO | 98(43.95%) |  |
| Distant metastases |  |  |
| YES | 0(0%) |  |
| NO | 223(100%) |  |
| AJCC disease stage |  |  |
| I+II | 153(68.61%) |  |
| III+IV | 70(31.39%) |  |
