# Supplementary material for: A panel of four genes accurately differentiates benign from malignant thyroid nodules
Source: J Exp Clin Cancer Res. 2016 Oct 28;35:169. doi: 10.1186/s13046-016-0447-3 (PMC5084448; doi:10.1186/s13046-016-0447-3)
Supplement: Additional file 2: Table S2. — Clinicopathologic characteristics of 146 benign thyroid lesions patients enrolled in the study. (DOCX 14 kb) [file 13046_2016_447_MOESM2_ESM.docx]

| Supplemental Table 2. Clinicopathologic characteristics of 146 benign thyroid lesions patients enrolled in the study. | |
| --- | --- |
| Characteristics | No. Patients (%) |
| Category |  |
| Adenoma | 38(26.03%) |
| Nodular goiter | 96(65.75%) |
| Lymphocytic thyroiditis | 10(6.85%) |
| Hyperthyroidism | 2(1.37%) |
| Age at diagnosis, y |  |
| Mean±SD | 50.68±12.51 |
| < 45 y | 45(30.82%) |
| ≥ 45 y | 101(69.18%) |
| Gender |  |
| Female | 119(81.51%) |
| Male | 27(18.49%) |
